# 

**Published:** 1879-06-20

**Authors:** 


### TABLE OF CONTENTS.

ORIGINAL AND SELECTED ARTICLES.

Croupus Pneumonia a Specific Infectious Disease, by A. C. Hobbs, M.D., of Indiana, 201. Cinchonia Alkaloid, by T. S. Lallerstedt, M.D., of Ga., 207. Diphtheria, by W. W. Carpenter, M.D., of California; Effects of Sudden Stoppage of Hypodermic Morphia, 209. Colden's Liebig's Liquid Extract of Beef, by W. A. Greene, M.D., of Macon, Ga., 211. The Prophylaxis of Puerperal Convulsions, 212. New Uses for Veratrum Viride, by M. E. Bishop, M.D., South Haven, Mich., 213. Jaborandi in Whooping-Cough, by E. A. De Cailhol, M.D., 215. Tracheotomy with the Thermo-Cautery, by P. S. Conner, M.D., 216.

ABSTRACTS AND GLEANINGS.

Lactopeptine; Bronchitis from Mouth Breathing, 218. Spinal Curvatures; Tubercular Ulcer of the Tongue, 219. Rules for Injecting Piles; Salicylate of Quinine as an Antipyretic, 220. Vaseline and Unguentum Vaselini Plumbicum in Skin Disease; Rheumatism of the Diaphragm, 221. First Insensibility from the Inhalation of Ether; Remedy for Dysentery, 222. Oxide of Zinc in Diarrhœa; Treatment of Infantile Paralysis; Epithelioma, 223. Fish-Bones in the Pharynx; A Forerunner of Death; Remedy for Sciatica, 224. Antidote to Arsenic; Hydrobromate of Quinia as an Antipyretic; Bismuth in Skin Affections; Chloral in Retention of Urine, 225. Treatment of Carbuncle by Blisters; Prevention of Fatal Accidents from Using Anæsthetics; Antidote for Arsenite of Soda, 226. Ovariotomy Superseded; Dichloride of Ethidene as an Anæsthetic; Salicylic Acid; Breech Presentations, 227. Relief of Pain from the Application of Sulphate of Copper; Hiccough; Operation for Phimosis; Action of Iodoform; Ergot and Sodium Bromide in Epilepsy, 228. Can One Have Typhoid Fever After Forty? Condition of the Tongue Valuable in the Diagnosis of Gastric Disorders; Differential Diagnosis between True Epilepsy and Hystero-Epilepsy; Carbolic Acid in Nasal Polypus, 229. Belladonna Hypodermically in Congestive Chills; Pyrogallic Acid in Psoriasis; Ergotine in Ophthalmia; Cataract, 230. Chloroform in Labor; Sodium Ethylate in Nævus, 217.

SCIENTIFIC ITEMS.

Camera Obscura; Rapidity of Thought in Dreaming, 231. A New Theory of Electricity; Gallium; Philippium; Gossamers; Longevity and Civilization; Alpha, 222.

PRACTICAL NOTES AND FORMULÆ.

Foul Breath; Worms, 233. Dysentery and Cholera Infantum, 234. Iodoform; Angina; Moles; Coryza; Toothache, 235. Hiccough; Vulcanized Rubber Nipples; Emulsio Expectorans; Mistura Bronchi; Castor Oil, 236.

EDITORIAL AND MISCELLANEOUS.

Southern Medical College; Correction; American Surgical Society; National Board of Health; Daily Dispatch; Convention of Medical Colleges, 237. Book Notices, 238.

